# mSphere of Influence: Where the Pathogen Proteins Are

**DOI:** 10.1128/mSphere.00365-21

**Published:** 2021-05-05

**Authors:** Aaron W. Reinke

**Affiliations:** a Department of Molecular Genetics, University of Toronto, Toronto, Ontario, Canada

**Keywords:** APEX, BioID, intracellular pathogens, microsporidia, protein localization

## Abstract

Aaron Reinke studies microsporidian evolution and how microsporidia interact with their hosts. In this mSphere of Influence article, he reflects on how the papers “A promiscuous biotin ligase fusion protein identifies proximal and interacting proteins in mammalian cells” (K. J. Roux, D. I. Kim, M. Raida, and B. Burke, J Cell Biol 196:801–810, 2012, https://doi.org/10.1083/jcb.201112098) and “Proteomic mapping of mitochondria in living cells via spatially restricted enzymatic tagging” (H.-W. Rhee, P. Zou, N. D. Udeshi, J. D. Martell, et al., Science 339:1328–1331, 2013, https://doi.org/10.1126/science.1230593) impacted his thinking on how to determine where proteins from intracellular pathogens are located within host cells.

## Abstract (precis)

Aaron Reinke studies microsporidian evolution and how microsporidia interact with their hosts. In this mSphere of Influence article, he reflects on how the papers “A promiscuous biotin ligase fusion protein identifies proximal and interacting proteins in mammalian cells” (K.

## COMMENTARY

Intracellular pathogens carry genes that encode a wide variety of different proteins that they use to control and exploit their hosts. These proteins include those that are secreted as well as proteins that can make contact with host proteins owing to localization to the plasma membrane of the pathogen. As I was starting my postdoctoral studies, I became fascinated with microsporidia, a large phylum of obligate intracellular parasites. Microsporidia have the smallest eukaryotic genomes and are extremely reliant on their hosts for resources (1). The types of proteins that they use to interface with their hosts were an outstanding question about microsporidian biology. At the time, unbiased approaches to determine protein localization relied upon biochemical purification of cellular organelles, which posed a hurdle to enlisting this strategy in host-pathogen studies, since separating intracellular pathogens from hosts is extremely challenging. Further, subcellular fractionation techniques suffer from both lack of sensitivity and specificity. Two related papers by Roux et al. (2) and Rhee et al. (3) described a novel approach of covalently modifying proteins with a chemical handle using enzymes that label proteins within discrete areas of intact cells, therein offering a solution to the problem of determining the cellular localization of proteins. After labeling, the cells could be disrupted, and the chemical handle could be used to identify proteins that resided in the target locations.

These two papers rely on conceptually related approaches but differ in their use of enzymes. The paper by Roux et al. employed BirA, an enzyme from Escherichia coli which specifically biotinylates acetyl coenzyme A (acetyl-CoA) carboxylase (2). Mutations in BirA had been previously shown to activate and release biotin, thus creating a cloud of reactive biotin in the immediate proximity of the enzyme. As a demonstration of an approach the authors named BioID, they fused this mutant version of BirA to lamin A which targeted the enzyme to the nuclear envelope. They expressed the fusion protein in human cells and the addition of exogenous biotin for 24 h resulted in robust biotinylation of known and previously unknown nuclear envelope-associated proteins. The paper by Rhee et al. exploited an engineered ascorbate peroxidase enzyme (APEX) from soybeans in an approach named spatially restricted enzymatic tagging (3). In the presence of hydrogen peroxide, APEX oxidizes a modified version of biotin containing a phenol group, resulting in the production of phenoxyl radicals which can covalently react with surface-exposed tyrosines on proteins in the immediate vicinity of the enzyme. The authors generated a fusion protein that targeted APEX to the mitochondrial matrix, expressed this protein in human cells, and labeled it with hydrogen peroxide and biotin-phenol for 1 min to profile mitochondrial proteins. Both set of authors then extracted, isolated, and identified biotinylated proteins using mass spectrometry. In this way, the teams identified many proteins known to localize to the organelle of interest, as well as novel proteins, that they confirmed by subsequent localization studies. Together, these papers described a powerful unbiased approach to profile proteins in specific subcellular compartments through the expression of genetically encoded enzymes. The promise of these papers was that the described techniques were very generalizable and could be applied to a wide range of biological questions.

During my postdoc, I set out to identify the proteins that microsporidia could use to directly manipulate and hijack host pathways. I first adapted spatially restricted enzymatic tagging in Caenorhabditis elegans to enable identification of proteins within different subcellular locations and tissues within the animal (4). Using the adapted technique, I then identified microsporidian proteins that were secreted from the pathogen or attached to the pathogen membrane, providing large-scale, and unbiased, experimental localization of pathogen proteins inside cells of a living animal host for the first time. The experimentally identified host-exposed microsporidian repertoire is enriched for proteins containing targeting signals, rapidly evolving proteins, and greatly expanded gene families. This work suggests that microsporidia use a large set of species-specific proteins as a common strategy to interact with their hosts (5).

Spatially restricted enzymatic tagging and BioID have now been used to determine the localization of proteins from a variety of intracellular protists, bacteria, and viruses. Examples include the identification of parasite proteins that localize to the parasitophorous vacuole membrane in Plasmodium berghei and Toxoplasma gondii (6, 7) and host proteins that localize to the vacuole membrane of Chlamydia trachomatis (8). Additionally, by tagging pathogen proteins with BirA or APEX, the cellular organelles and interacting partners of these proteins can be revealed, as shown with several Salmonella effector proteins or with all of the proteins encoded by the Zika and severe acute respiratory syndrome coronavirus 2 (SARS-CoV-2) viruses (9–11). New improved versions of both BioID and APEX have been developed, and it is expected that this enzyme-based labeling strategy will be applied to a wide variety of pathogens in the future, further elucidating where the pathogen proteins are (12, 13).
